# *In Vitro* and *In Vivo* Antiangiogenic Activity of Caged Polyprenylated Xanthones Isolated from *Garcinia hanburyi* Hook. f.

**DOI:** 10.3390/molecules181215305

**Published:** 2013-12-11

**Authors:** Jianhong Yang, Shichao He, Shucai Li, Ronghong Zhang, Aihua Peng, Lijuan Chen

**Affiliations:** State Key Laboratory of Biotherapy, West China Hospital, West China Medical School, Sichuan University, Chengdu 610041, Sichuan, China

**Keywords:** *Garcinia hanburyi*, caged polyprenylated xanthone, gambogic acid, cytotoxicity, angiogenesis, zebrafish

## Abstract

Eleven known caged polyprenylated xanthones **1**–**11** were isolated from the resin of *Garcinia hanburyi* Hook. f., and their structures were identified by their MS, NMR and UV spectra. These xanthones showed significant cytotoxicities against four human cancer cell lines (HeLa, A549, HCT-116, and HepG-2) and strong inhibition against the proliferation of the HUVEC cell line *in vitro* by the MTT method. Furthermore, in an *in vivo* zebrafish model, xanthones **3** (morellic acid), **7** (gambogenin) and **9** (isogambogenic acid) showed comparable antiangiogenic activities with less toxicities than xanthone **1** (gambogic acid), as evaluated by death and heart rates of treated zebrafish. Xanthone **7** exhibited antiangiogenic activity with no toxicity at concentrations ranging from 8 µM to 16 µM. Meanwhile, xanthones **1**, **3**, **7** and **9** strongly inhibited the migration of HUVEC at a low concentration of 0.5 µM in HUVEC cell migration assay *in vitro*. Taken together, these findings strongly suggest that xanthone **7** might be a novel angiogenesis inhibitor.

## 1. Introduction

Angiogenesis plays a critical role in tumor progression [1,2]. Angiogenesis, the growth of new microvessels from existing vasculature, is a tightly regulated process that mainly involves endothelial cell proliferation, migration, and organization into capillaries [3]. Therefore, inhibition of tumor angiogenesis has been a promising strategy in the development of novel anticancer therapy.

*Garcinia hanburyi* Hook. f., a plant belonging to the Guttiferae family, is a small tree distributed throughout Thailand, Cambodia, India, and the southern part of China. Its resin is used as a dye and folk medicine for its potent purgative effects, and in the treatment of infected wounds [4]. It had been developed in the 1970s as an anti-tumor drug via intravenous injection in China for clinical testing [5]. Gambogic acid (GA), a caged polyprenylated xanthone, is a natural product isolated from the resin of *Garcinia hanburyi* trees in southeastern Asia [6]. Recent studies from several laboratories have demonstrated that this natural product possesses potent anticancer activity, both *in vitro* and *in vivo* [7,8,9]. The potent anticancer activity of GA is mainly attributed to its activation of the impaired apoptosis pathways in cancerous cells via down-regulation of telomerase [9,10,11]. Moreover, GA is a potent angiogenesis inhibitor, which inhibits angiogenesis through suppression of vascular endothelial growth factor (VEGF)-induced tyrosine phosphorylation of KDR/Flk-1 and GA showed antiangiogenesis activity *in vitro* and *in vivo* [12,13]. However, compared with other caged polyprenylated xanthones which are structural analogues of GA, GA exhibited higher toxicity and there are few reports about the antiangiogenic activity and toxicity of GA structural analogues, so this led us to question whether other caged polyprenylated xanthones could exert similar antiangiogenesis activities with less toxicities.

The teleost zebrafish (*Danio rerio*) is an elegant, small tropical fish with a short generation time [14]. It has rapidly emerged as an ideal vertebrate model to study biological processes in adults as well as during development. Zebrafish offer a powerful drug screening platform because of their transparent embryos, the ease of embryo maintenance, the simplicity of the experimental techniques, and the cost-effective and truly quantitative assay formats [15]. Moreover, their transparency becomes even more useful when fluorescent markers are used to label specific populations of cells (e.g., endothelial cells and cranial motor neurons) [16], making zebrafish is especially suitable for identification of angiogenesis inhibitors, since the development of blood vessels in early embryos is well characterized and easily monitored [17].

In our research on the resin of *Garcinia hanburyi*, eleven known caged polyprenylated xanthones **1**–**11** were isolated, including the known GA as a major active constituent. To our knowledge, apart from GA, only isogambogenic acid has been examined systematically for antiangiogenesis activity [18]. In this study, we report the isolation and structural elucidation of xanthones **1**–**11**, as well as the assessment of their cytotoxicities against four human cancer cell lines (HeLa, A549, HCT-116, and HepG-2) and their inhibitory activity against the proliferation of the HUVEC cell line. Through zebrafish antiangiogenic screening in zebrafish embryos, we have identified three antiangiogenic caged polyprenylated xanthones (xanthones **3**, **7** and **9**) with less toxicity, compared to GA. The subsequent HUVEC migration assay demonstrated the antiangiogenic activities of the three xanthones above.

## 2. Results and Discussion

**Figure 1:**
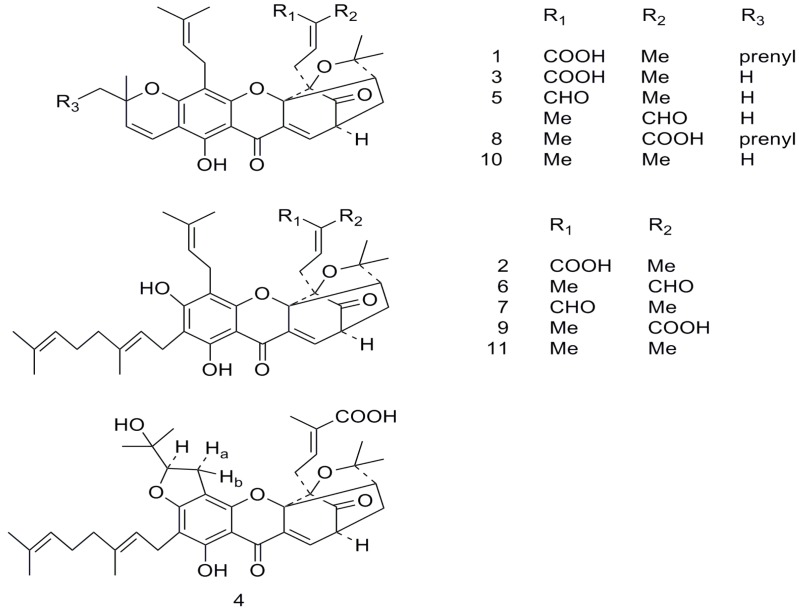
The structures of caged polyprenylated xanthones **1**–**11** isolated from *G. hanburyi.*

Eleven known caged polyprenylated xanthones **1**–**11** (Figure 1) were separated from the dried resin of *Garcinia hanburyi.* These xanthones were identified as gambogic acid (**1**) [19], gambogenic acid (**2**) [20], morellic acid (**3**) [21], gambogenific acid (**4**) [22], morellin isomers **5** [23], isogambogenin (**6**) [20], gambogenin (**7**) [20], isogambogic acid (**8**) [19], isogambogenic acid (**9**) [5], desoxymorellin (**10**) [24], and desoxygambogenin (**11**) [20], respectively, by comparison of their spectroscopic data with those of the appropriate literatures. Of them, the compounds **5** are two isomers, and could not be further separated in our study, so the isomers were tested as one compound in our subsequent experiments.

As shown in Table 1, all the tested xanthones **1**–**11** exhibited potent cytotoxicities against four human cancer cell lines.

**Table 1:** Cytotoxicity of caged polyprenylated xanthones 1–11 against four cultured human cancer cell lines and HUVEC cell line in the MTT assay.

Xanthone	IC_50_ (μM)				
	HeLa	A549	HCT-116	HepG-2	HUVEC
1	1.59	1.55	0.64	0.78	0.72
2	11.50	4.76	4.76	6.35	4.12
3	13.78	14.23	8.89	13.34	6.14
4	7.58	11.30	4.49	10.21	2.48
5	5.33	6.43	2.76	5.97	2.39
6	4.07	4.88	1.47	2.44	2.12
7	3.26	4.88	1.30	6.51	1.04
8	4.77	2.86	1.59	6.37	0.91
9	6.35	12.69	11.74	6.35	1.73
10	2.60	2.05	1.20	1.00	0.90
11	2.95	1.80	1.35	1.15	1.60

IC_50_: the concentration of the compound that caused a 50% inhibition of cell growth.

Xanthone **1** (GA) showed the highest cytotoxicity against HeLa, A549, HCT-116, and HepG-2 human cancer cell lines with IC_50_ values of 1.59, 1.55, 0.64, and 0.78 μM, respectively. Xanthone **10** (desoxymorellin) and xanthone **11** (desoxygambogenin) also displayed significant cytotoxicities, second only to xanthone **1**. Xanthones **1**, **7**, **8**, and **10** exhibited comparable anti-proliferative activity against the HUVEC cell line (IC_50_ = 0.72, 1.04, 0.91, and 0.90 μM, respectively).

**Figure 2:**
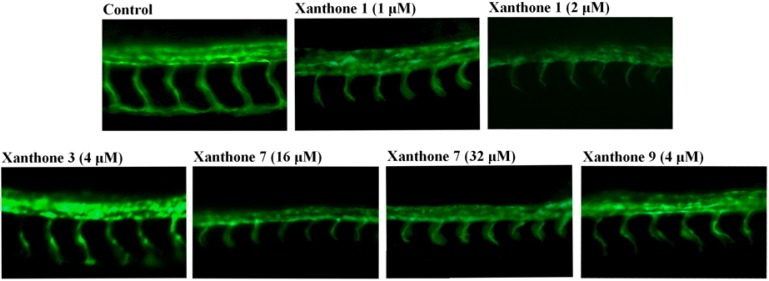
Antiangiogenic effect of xanthones **1**, **3**, **7** and **9** in zebrafish embryos. (magnification 400×) The results shown are representative of three independent experiments.

Next, we examined the effect of the xanthones **1**–**11** on embryonic angiogenesis. Table 2 shows the antiangiogenic phenotypes of different concentration gradients of the xanthones **1**–**11** in the zebrafish embryos. Among them, xanthones **1**, **3**, **7**, and **9** displayed antiangiogenic activity to zebrafish embryos with different concentrations varying from 1 μM to 16 μM, but the other xanthones were inactive. As shown in Figure 2, xanthone **1** (GA), a known antiangiogenic compound, was taked as a positive control and significantly exhibited the growth of angiogenic intersegmental vessels (ISVs) in embryos with 1 μM and 2 μM for 24 h. Most of the ISVs were incompletely formed compared with the embryo water-treated control. Xanthones **3**, **7** and **9** also exhibited comparable inhibitory effects on ISV formation to GA with higher concentrations at 4 μM, 32 μM, 4 μM, respectively.

**Table 2:** Antiangiogenic phenotype of caged polyprenylated xanthones **1**–**11** in zebrafish embryos. The results shown are representative of three independent experiments.

Xanthone	Antiangiogenic Phenotype					
	1 μM	2 μM	4 μM	8 μM	16 μM	32 μM
1	√	√				
2	×	×				
3	×	×	√			
4	×	×	×	×		
5	×	×	×			
6	×	×	×	×	×	×
7	×	×	×	×	√	√
8	×	×	×	×		
9	×	×	√			
10	×	×	×	×		
11	×	×	×	×		

**√**: Exhibit antiangiogenic activity; ×: inactive.

According to the death and heart rates of drug-treated zebrafish embryos summarized in Table 3, xanthones **1**, **3** and **9** caused the death of all zebrafish embryos at the highest concentrations at 4, 8 and 8 μM, respectively. However, xanthone **7** didn’t cause any zebrafish embryo deaths at the antiangiogenic concentrations of 16 μM and 32 μM. Meanwhile, xanthones **1**, **3**, **7** and **9** decreased the heart rates of zebrafish embryos as their concentrations increased to the right levels (Table 4). Together, these data indicated that xanthone **7**, which demonstrated potent antiangiogenic activity and no toxicity at appropriate concentrations in zebrafish embryos, has potential for development as an antiangiogenic agent.

**Table 3:** Death rates of caged polyprenylated xanthones **1**–**11** in zebrafish embryos. Zebrafish embryos treated with embryo water were used as control and showed no death (0). The results shown are representative of three independent experiments.

Xanthone	Death Rate					
	1 μM	2 μM	4 μM	8 μM	16 μM	32 μM
1	0	1/8	8/8			
2	0	0	8/8			
3		0	0	8/8		
4		0	0	0		
5		0	0	8/8		
6				0	1/8	4/8
7				0	0	0
8		0	1/8	6/8		
9		0	0	8/8		
10	0	0	0	0		
11	0	0	0	0		

**Table 4:** Heart rates of caged polyprenylated xanthones **1**–**11** in zebrafish embryos. Zebrafish embryos treated with embryo water were used as control and showed the heart rate: 21 per 10 s. The results shown are representative of three independent experiments.

Xanthone	Heart Rate (per 10 s)					
	1 μM	2 μM	4 μM	8 μM	16 μM	32 μM
1	8	3	0			
2	21	18	0			
3		8	6	0		
4		21	17	13		
5		13	10	8		
6				17	14	8
7				6	6	5
8		17	13	6		
9		8	5	0		
10	20	17	16	16		
11	21	21	21	20		

**Figure 3:**
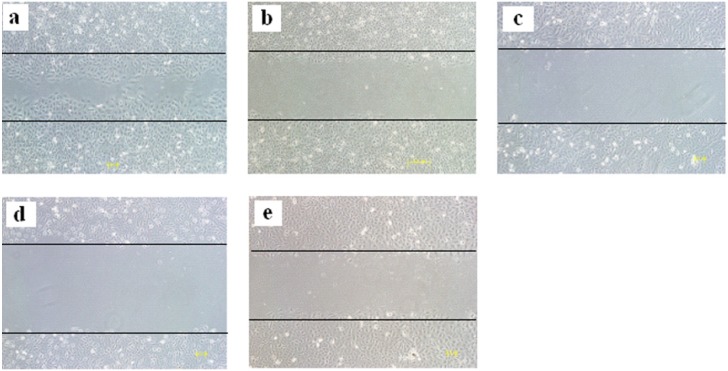
Effects of xanthones on HUVEC migration in wound migration assays. (**a**): Control, medium alone. (**b**) 0.5 μM xanthone **1**. (**c**) 0.5 μM xanthone **3**. (**d**) 0.5 μM xanthone **7**. (**e**) 0.5 μM xanthone **9**. The results shown are representative of three independent experiments.

Endothelial cell migration is essential to angiogenesis. Here, xanthones **1**, **3**, **7** and **9** which displayed antiangiogenic activities in zebrafish embryos were chosen to determine the effects on HUVEC migration. As shown in Figure 3, all four xanthones mentioned above exhibited remarkable inhibition on the migration of HUVEC at a low concentration of 0.5 μM.

## 3. Experimental

### 3.1. Plant Material

The gamboge resin of *Garcinia hanburyi* was purchased in Chengdu, Sichuan Province, China, in 2009 and indentified by Dr. Yanfang Li (Department of Pharmaceutical Engineering, College of Chemical Engineering, Sichuan University). The voucher specimen was deposited at the State Key Laboratory of Biotherapy, Sichuan University, Chengdu, China.

### 3.2. Isolation

The dried resin of *Garcinia hanburyi* (200 g) was powdered and extracted with acetone (3×1 L) under reflux for three times (2 h. each time).The acetone solution was concentrated under reduced pressure to give a brown-yellow gum (90 g), which was fractionated by silica gel column chromatography with a gradient of petroleum ether–acetone (90:10 to 0:100) as eluent to give eight fractions. Each fraction was subjected to various silica gel and reversed phase C-18 chromatography procedures to yield the eleven known caged polyprenylated xanthones **1**–**11**.

### 3.3. MTT Assay

The xanthones **1**–**11** were tested for cytotoxicity against HeLa, A549, HCT-116, and HepG-2 human cancer cell lines and inhibition on the proliferation of the HUVEC cell line by using the MTT assay according to the described protocols [25].

### 3.4. Antiangiogenic Activity Assay on Blood Vessel Formation in Zebrafish Embryos

A transgenic zebrafish line, Tg(flk1:EGFP), with the endothelial- specific flk1 promoter directing enhanced green fluorescent protein (EGFP) expression in all endothelial cells of the vasculature was used [26]. Adult zebrafish were maintained at 28.5 °C in a recirculating aquaculture system. Zebrafish embryos were generated by natural pair-wise mating, collected in the morning and raised at 28.5 °C in embryo water (0.2 g/L of Instant Ocean Salt in distilled water). At 6 h post-fertilization (hpf), the embryos were sorted for viability and developmental stage (shield stage). The embryos were firstly placed in 24-well plates in 1 mL of embryo water with eight embryos per well. Then, the xanthones, at various concentrations, were added to embryo water of each well from the shield stage (about 6 h). Because the fish embryo receives nourishment from an attached yolk ball for the duration of the experiment, no additional maintenance was required during the duration of the experiments. After treatment with the drugs for 24 h, the embryos were anesthetized with 0.05% 2-phenoxyethanol in embryo water. One to three embryos were placed on carry sheet glass and each embryo was examined for the presence of ectopic vessels in the subintestinal vessel plexus (SIV) as an indication of antiangiogenic effect. Photographs were taken under a fluorescence microscope.

### 3.5. HUVEC Wound Migration Assay

The wound migration assay was performed as previously described [27]. HUVECs were seeded at a plating density of 2 × 10^5^ per well in 6-well plates which had been coated with 1% gelatin, cells grown in DMEM medium with 10% FBS overnight. After reaching 100% confluence, the cells were then gently scraped with a plastic tip to produce a wound area. After wounding cells were incubated with fresh DMEM medium with xanthones **1**, **3**, **7** and **9** at a concentration of 0.5 μM, the migration and cell movement throughout the wound area was examined after 24 h.

## 4. Conclusion

In conclusion, we demonstrated that xanthones **3**, **7** and **9** showed strong strong inhibitory activity against HUVEC migration in HEVEC migration assay *in vitro* and antiangiogenic activities with less toxicities than GA in zebrafish embryos *in vivo*. Especially xanthone **7** with the lowest toxicity, might serve as s potential angiogenesis inhibitor and anticancer drug.
